# Cumulative intravenous fluid volume in the first 24 hours and risk of respiratory deterioration in children hospitalized with community acquired pneumonia

**DOI:** 10.3389/fped.2026.1780766

**Published:** 2026-04-10

**Authors:** Zhu Zhu

**Affiliations:** 1 Department of Pediatrics, The First People’s Hospital of Yuhang District, Hangzhou, Zhejiang, China; 2 Department of Pediatrics, The First Affiliated Hospital Zhejiang University School of Medicine Liangzhu Branch, Hangzhou, Zhejiang, China

**Keywords:** community-acquired pneumonia, fluid-to-maintenance ratio, intravenous fluids, pediatrics, real-world study, respiratory deterioration

## Abstract

**Background:**

Intravenous (IV) fluids are frequently administered to children hospitalized with community-acquired pneumonia (CAP), yet excessive early IV volume may worsen gas exchange in inflamed lungs. We evaluated whether cumulative IV fluid exposure during the first 24 h was associated with subsequent respiratory deterioration.

**Methods:**

We conducted a single-center observational study using electronic health record data and a prespecified 24-hour landmark design. Early IV fluid exposure was quantified as the fluid-to-maintenance ratio (FMR), defined as total IV volume in 0–24 h divided by predicted 24-hour maintenance volume using Holliday-Segar and categorized into quartiles. The primary outcome was respiratory deterioration between 24 and 72 h, defined as escalation to high-flow nasal cannula, noninvasive ventilation, or invasive mechanical ventilation and/or ICU transfer for respiratory support. Associations were assessed using multivariable logistic regression, restricted cubic splines, overlap weighting for Q4 vs. Q1–Q3, and prespecified sensitivity/subgroup analyses.

**Results:**

Overall, 243 developed respiratory deterioration. Event rates increased across FMR quartiles (3.1%, 6.1%, 9.6%, 21.2%). Each 0.5-unit increase in FMR was associated with higher adjusted odds of deterioration (adjusted OR 1.45, 95% CI 1.28–1.65; *p* < 0.001). Compared with Q1, Q4 had higher adjusted odds (adjusted OR 3.56, 95% CI 2.08–6.10; *p* < 0.001), supported by overlap-weighted analysis (Q4 vs. Q1-Q3, OR 2.01, 95% CI 1.29–3.13). Spline modeling showed progressively increasing risk at higher FMR. Results were robust in most sensitivity analyses. In an early-response sensitivity analysis incorporating first 0–6 h fluid front-loading and age-standardized physiological response, the association attenuated but remained significant (adjusted OR 1.27, 95% CI 1.10–1.47; *p* = 0.001). A complementary repeated-measures 0–6 h time-slope analysis using all available vital-sign recordings yielded similar results (adjusted OR 1.29, 95% CI 1.12–1.49; *p* = 0.001) (adjusted OR 1.31, 95% CI 1.14–1.50; *p* < 0.001). In the complete-case procalcitonin (PCT) subset, the estimate attenuated after additional adjustment for log(PCT) (adjusted OR 1.18, 95% CI 0.98–1.43).

**Conclusions:**

Higher IV fluid exposure relative to maintenance during the first 24 h was associated with increased risk of subsequent respiratory deterioration in children hospitalized with CAP. These findings support prospective validation and evaluation of maintenance-aware IV fluid approaches.

## Introduction

Pediatric community-acquired pneumonia (CAP) remains a significant health burden, being the leading cause of infectious disease deaths in children under five globally (1, 2). The disease is primarily caused by viral pathogens, although bacterial infections, particularly by *Streptococcus pneumoniae*, are also common (3, 4). The introduction of pneumococcal vaccines has reduced bacterial CAP incidence, yet the overall disease burden remains high, especially in developing countries (1). Hospitalization for CAP is often necessary when children present with severe symptoms such as hypoxia or respiratory distress, and the management typically involves supportive care, including oxygen therapy, antimicrobials, and intravenous (IV) fluids (5, 6). The standard treatment for hospitalized children includes narrow-spectrum antibiotics like ampicillin or penicillin G, especially for those who are fully immunized (5, 7). However, differentiating between viral and bacterial CAP is challenging, often leading to unnecessary antibiotic use (5, 8). Implementation of clinical pathways has shown to reduce inappropriate antibiotic use, such as ceftriaxone, and improve adherence to recommended practices (3, 9). Additionally, emerging diagnostic tools like procalcitonin (PCT) testing and metagenomic next-generation sequencing (mNGS) show promise in improving diagnostic accuracy and guiding targeted therapy (1, 4).

The biological plausibility of why fluids may worsen the respiratory course, particularly in pediatric acute respiratory distress syndrome (PARDS), is supported by several studies that highlight the detrimental effects of fluid overload. Excessive intravenous fluid administration can lead to increased extravascular lung water, reduced lung compliance, and impaired oxygenation, especially in the presence of inflamed alveolar-capillary interfaces. This is particularly concerning in conditions like community-acquired pneumonia (CAP) associated with syndrome of inappropriate antidiuretic hormone secretion (SIADH) and hyponatremia (10–12). A positive cumulative fluid balance after the fourth day of PARDS was linked to higher mortality and lower probability of extubation. Fluid overload in children post-hematopoietic cell transplantation with PARDS was strongly associated with higher oxygenation impairment and increased mortality (13). The pathophysiology involves damage to the endothelial glycocalyx during systemic inflammatory response syndrome, leading to fluid extravasation and interstitial edema (14). Despite the clear association between fluid overload and adverse outcomes, there is a notable evidence gap in pediatric data, as many studies do not precisely quantify cumulative 0–24 h IV volume or ensure correct temporality, often mixing diagnoses and failing to examine nonlinear thresholds and effect modification (11, 15). This underscores the need for age-specific fluid management protocols to improve outcomes in pediatric patients with PARDS (12). An additional unresolved question is whether higher early IV volume mainly reflects greater presenting severity and incomplete early response to initial treatment rather than a potentially modifiable exposure itself.

Therefore, we aimed to determine whether higher cumulative IV fluid volume within the first 24 h of hospitalization predicts respiratory deterioration occurring after hour 24 in children admitted with CAP. We hypothesized that greater early IV fluid exposure would be associated with higher risk of post-24-hour respiratory escalation and that risk would increase in a dose-dependent manner, particularly among children with physiologic or radiographic features suggesting reduced fluid tolerance.

## Methods

### Study design and setting

We performed a single-center, non-interventional, real-world observational study at The First People's Hospital of Yuhang District using routinely collected electronic health record (EHR) data. We prespecified a 24-hour landmark design in which cumulative IV fluid exposure was defined from admission time (T0) to T0 + 24 h, and respiratory deterioration outcomes were assessed only after the landmark, within 24–72 h. Clinical decisions regarding fluids and respiratory support were made by treating clinicians independent of the study. This study was conducted in accordance with the Declaration of Helsinki. The protocol was reviewed and approved by the Ethics Committee of The First People's Hospital of Yuhang District, which waived the requirement for informed consent because this was a retrospective analysis of de-identified routinely collected data and posed no more than minimal risk to participants. All data were anonymized prior to analysis.

### Participants and case definition

We included hospitalized children with CAP, defined as an acute infection of the lung parenchyma acquired outside the hospital, with symptom onset before admission and a clinical diagnosis established at presentation or within the first 48 h of hospitalization (to distinguish CAP from hospital-acquired pneumonia). Case identification was supported by clinician documentation of pneumonia and pneumonia-directed treatment. Radiographic findings consistent with pneumonia were used when available.

We excluded encounters meeting criteria for hospital-acquired or ventilator-associated pneumonia, primary aspiration/chemical pneumonitis, and conditions with distinct fluid protocols (e.g., dialysis-dependent kidney failure, diabetic ketoacidosis, major burns). We also excluded admissions without sufficient infusion documentation to compute cumulative 0–24-hour IV volume. For the primary landmark cohort, we excluded children who experienced respiratory deterioration within the first 24 h or were discharged before 24 h to ensure all analyzed patients were event-free and at risk at the landmark time.

### Data sources and variable ascertainment

We extracted demographics, comorbidities, vital signs, oxygen therapy, diagnoses, and disposition from the EHR/HIS, laboratory values from the laboratory information system, imaging phenotypes from radiology reports, and IV fluid volumes from nursing infusion/eMAR records.

Baseline physiologic status was defined using the earliest documented SpO_2_ and oxygen delivery category at presentation (room air, low-flow oxygen, or face mask). Baseline imaging phenotypes (multilobar involvement, pleural effusion) were abstracted from the first chest radiograph obtained early in hospitalization (and from ultrasound/CT when performed to further evaluate pleural fluid). Baseline laboratory covariates were defined using the earliest available values recorded near presentation within routine clinical care (sodium, albumin, CRP). Because some laboratory values could be influenced by early therapy, we interpret them as markers of early illness phenotype and performed prespecified sensitivity analyses to test robustness. Procalcitonin (PCT) was recorded when ordered as part of routine care and evaluated in a complete-case sensitivity analysis due to non-universal testing. Baseline sodium was intended to capture early illness phenotype at presentation rather than subsequent iatrogenic changes from maintenance-fluid tonicity; nevertheless, isotonic maintenance fluid use was recorded and included as an adjustment covariate. For early-response analyses, we extracted all documented heart rate, respiratory rate, systolic blood pressure, diastolic blood pressure, SpO_2_, and oxygen delivery observations within 6 h of admission. Heart rate and respiratory rate were converted to age-standardized z scores using published pediatric reference centiles, and blood pressure was standardized to age-appropriate pediatric reference tables (16, 17). We then derived two exploratory early-response specifications: (1) an anchored baseline-to-nearest-6-hour model using change in vital-sign z scores, absolute change in SpO_2_, change in oxygen category, and the proportion of 24-hour IV volume delivered in the first 6 h; and (2) a repeated-measures first-6-hour model using all available observations to estimate patient-specific per-hour slopes for vital-sign z scores and SpO_2_, together with early oxygen-category trajectory.

### Exposure definition: cumulative IV fluids in the first 24 h

The primary exposure was cumulative IV fluid volume delivered from T0 to T0 + 24 h, calculated by summing all documented IV maintenance fluids (including dextrose-containing solutions) and IV bolus fluids recorded in nursing infusion documentation during the first 24 h. IV fluid included crystalloids used for maintenance and/or bolus therapy (e.g., 0.9% sodium chloride–based solutions, balanced crystalloids such as lactated Ringer's/Plasma-Lyte, and dextrose-containing saline solutions). When colloids (e.g., albumin) were administered, their volumes were included if documented.

Exposure was expressed as a fluid-to-maintenance ratio (FMR), defined as total IV volume in the first 24 h divided by predicted 24-hour maintenance requirement estimated using the Holliday–Segar 100/50/20 method. Because predicted maintenance may not represent a physiologic “target” for acutely ill children, we used FMR primarily as a normalization metric for comparing exposure across ages and body sizes.

### Outcome definition: respiratory deterioration after the 24-hour landmark

The primary outcome was respiratory deterioration occurring between 24 and 72 h after admission, defined as escalation to advanced respiratory support (initiation of high-flow nasal cannula, noninvasive ventilation, or invasive mechanical ventilation) and/or ICU transfer for respiratory support during the post-landmark window. Outcomes were ascertained using time-stamped respiratory support orders/device documentation, ventilator records, and ICU transfer timestamps. Only the first qualifying deterioration event per child was counted. Children without deterioration by 72 h were classified as non-events for the primary fixed-window analysis.

### Covariates

We prespecified covariates plausibly related to both fluid prescribing and respiratory deterioration risk, prioritizing variables available at presentation. The adjusted model included age, sex, baseline oxygen category, multilobar involvement, pleural effusion, log-transformed CRP, hyponatremia (sodium <135 mmol/L), hypoalbuminemia (albumin <35 g/L), and major comorbidities (asthma/recurrent wheeze, congenital heart disease, neurologic disease, and immunocompromise). Because early hemodynamic instability may influence fluid administration and subsequent deterioration risk, we included vasoactive agent use within the first 24 h as a marker of early resuscitation context and assessed robustness by excluding these encounters in sensitivity analyses. Isotonic maintenance fluid use (yes/no) was recorded to describe maintenance strategy and included as an adjustment variable. To better separate baseline severity from later clinical evolution, the primary model emphasized covariates available at presentation rather than interval post-exposure changes. Because blood pressure data were less uniformly available across the complete landmark cohort and the primary model was prespecified to prioritize widely available presentation covariates, blood pressure was incorporated in early-response sensitivity analyses but was not added to the primary adjusted model.

To partially account for baseline cardiac dysfunction that may influence fluid tolerance and respiratory course, documented congenital heart disease was included *a priori* in the comorbidity set used for adjustment.

Isotonic maintenance fluids were operationally defined as 0.9% sodium chloride–based solutions or balanced isotonic crystalloids (e.g., lactated Ringer's/Plasma-Lyte), with or without dextrose, consistent with pediatric maintenance IV fluid guidance (18).

### Statistical analysis

Baseline characteristics were summarized by FMR quartiles using mean (SD) or median [IQR] for continuous variables and *n* (%) for categorical variables. The primary association between early IV fluids and respiratory deterioration (24–72 h) was evaluated using logistic regression, reporting ORs and 95% CIs for FMR per 0.5 increase and for quartiles with Q1 as the reference, and an ordinal trend test was performed by modeling quartile as an ordered variable. We applied Benjamini–Hochberg false discovery rate (FDR) correction to adjusted *p* values. All analyses were conducted in R version 4.4 (R Foundation for Statistical Computing, Vienna, Austria) using glm() for logistic regression, rms::lrm() with rcs() for restricted cubic splines, overlap weighting based on the estimated propensity score, robust variance estimators from sandwich, and p.adjust(method = “BH”) for FDR correction.

To evaluate nonlinearity, we fit an adjusted restricted cubic spline logistic model for FMR (4 degrees of freedom) and plotted adjusted ORs with 95% CIs relative to FMR = 1.0. To improve covariate balance for the high-exposure comparison, we conducted an overlap-weighted analysis comparing Q4 vs. Q1–Q3 by estimating a propensity score for being in Q4 using the same baseline covariates as the primary adjusted model and applying overlap weights (1−PS for Q4 and PS for Q1–Q3) in a weighted logistic regression. We assessed covariate balance before and after weighting using standardized mean differences and used robust (sandwich) variance estimators for weighted models.

#### Subgroup and sensitivity analyses

Effect modification was assessed by adding interaction terms between continuous FMR (per 0.5 increase) and prespecified subgroups (hyponatremia, hypoalbuminemia, pleural effusion, baseline oxygen use, and age <1 year), reporting stratum-specific adjusted ORs and interaction *P* values with FDR correction. Sensitivity analyses tested alternative outcome windows (24–48 h and 24 h to 7 days), excluded children receiving vasoactive agents within 24 h, restricted analyses to children without documented IV bolus in the first 24 h, and performed a complete-case analysis among children with measured PCT that added log(PCT) to the adjusted model. To further examine whether higher IV exposure primarily represented greater illness severity, more rapid early fluid delivery, or poor early response to treatment, we performed two additional exploratory sensitivity analyses. The first was an anchored baseline-to-nearest-6-hour model among children with analyzable early-response data, additionally adjusting for change in heart rate, respiratory rate, systolic blood pressure, and diastolic blood pressure z scores, absolute change in SpO_2_, change in oxygen category, and the proportion of 24-hour IV volume delivered in the first 6 h. The second used all available vital-sign recordings within 6 h to derive patient-specific per-hour slopes for these z scores and SpO_2_ and included early oxygen-category trajectory, thereby avoiding restriction to children with a single approximately 6-hour observation.

## Results

### Study cohort

After exclusion of children not eligible for the analysis, the final cohort contained 243 children experienced respiratory deterioration during 24–72 h (escalation to HFNC/NIV/IMV and/or ICU transfer for respiratory support), yielding an event rate of 10.0% (Supplementary Figure S1).

### Distribution of early IV fluid exposure

**Figure 1:**
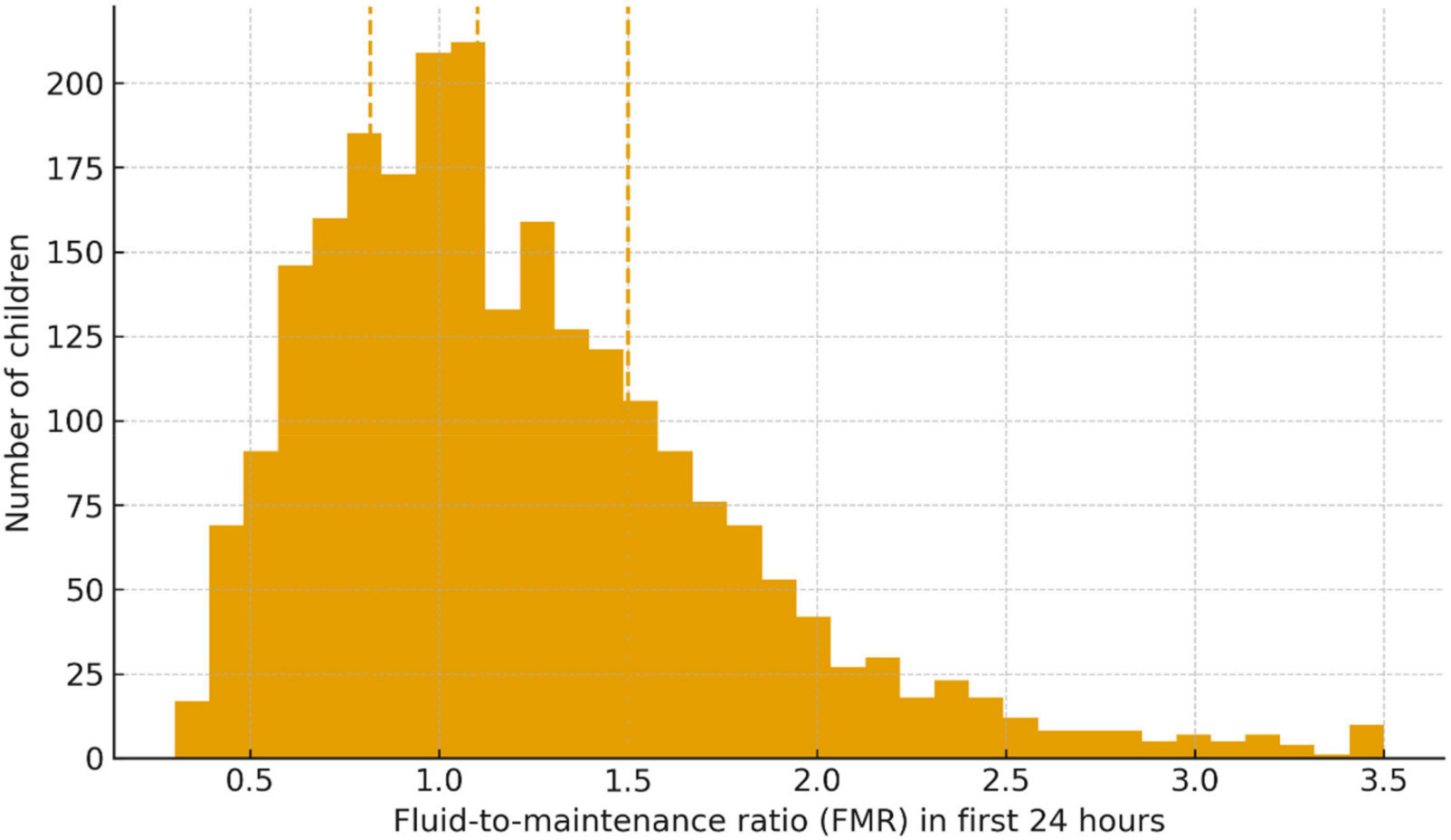
Distribution of 24-hour IV fluid exposure. Histogram of the fluid-to-maintenance ratio (FMR) in the first 24 h. Dashed vertical lines denote the 25th/50th/75th percentiles (quartile cut-points).

Early IV fluid exposure varied substantially. FMR quartile medians ranged from 0.66 (Q1) to 1.82 (Q4), and mean (SD) 24-hour IV volume increased from 51.3 (14.8) mL/kg in Q1 to 169.3 (50.6) mL/kg in Q4 (Table 1). The overall FMR distribution was right-skewed (Figure 1).

**Table 1:** Baseline characteristics by 24 h IV fluid exposure by FMR quartiles.

Characteristic	Q1 (lowest)	Q2	Q3	Q4 (highest)	*p* value
FMR ratio	0.66 [0.55, 0.75]	0.97 [0.89, 1.04]	1.29 [1.20, 1.38]	1.82 [1.65, 2.16]	
IV volume in first 24 h, mL/kg	51.3 (14.8)	79.0 (18.0)	106.5 (24.4)	169.3 (50.6)	
Age					<0.001
Age <1y	115 (18.9%)	169 (27.8%)	183 (30.1%)	251 (41.3%)	
Age 1–5y	308 (50.7%)	274 (45.1%)	266 (43.8%)	236 (38.8%)	
Age >5y	185 (30.4%)	164 (27.0%)	158 (26.0%)	121 (19.9%)	
Male	335 (55.1%)	327 (53.9%)	329 (54.2%)	360 (59.2%)	0.213
Weight, kg	17.6 (9.1)	16.8 (9.4)	16.5 (9.5)	14.5 (8.8)	<0.001
Baseline SpO_2_, %	96.6 (2.0)	96.3 (2.1)	95.7 (2.4)	94.6 (2.7)	<0.001
Baseline oxygen					<0.001
Room air	488 (80.3%)	456 (75.1%)	376 (61.9%)	255 (41.9%)	
Low-flow O_2_	107 (17.6%)	130 (21.4%)	185 (30.5%)	218 (35.9%)	
Face mask	13 (2.1%)	21 (3.5%)	46 (7.6%)	135 (22.2%)	
Multilobar involvement	153 (25.2%)	213 (35.1%)	239 (39.4%)	299 (49.2%)	<0.001
Pleural effusion	43 (7.1%)	69 (11.4%)	74 (12.2%)	103 (16.9%)	<0.001
CRP, mg/L	18.0 [10.8, 29.9]	19.7 [12.2, 32.6]	22.5 [13.6, 36.2]	23.7 [14.2, 39.5]	<0.001
Sodium, mmol/L	136.8 (2.8)	136.8 (2.9)	136.5 (3.0)	136.2 (3.0)	0.006
Hyponatremia (Na <135), *n* (%)	161 (26.5%)	153 (25.2%)	191 (31.5%)	211 (34.7%)	<0.001
Albumin, g/L	39.8 (4.7)	39.7 (4.6)	39.3 (4.9)	38.2 (4.9)	<0.001
Hypoalbuminemia (<35 g/L), *n* (%)	100 (16.4%)	96 (15.8%)	116 (19.1%)	149 (24.5%)	<0.001
Isotonic maintenance fluid, *n* (%)	439 (72.2%)	454 (74.8%)	455 (75.0%)	466 (76.6%)	0.356
Bolus given in first 24 h, *n* (%)	62 (10.2%)	92 (15.2%)	134 (22.1%)	287 (47.2%)	<0.001
Vasoactive use in first 24 h, *n* (%)	5 (0.8%)	12 (2.0%)	27 (4.4%)	76 (12.5%)	<0.001

### Baseline characteristics by exposure quartile

Higher FMR quartiles were associated with younger age and greater baseline respiratory severity (Table 1). Compared with Q1, Q4 contained a larger proportion of infants <1 year (41.3% vs. 18.9%), had lower mean weight (14.5 vs. 17.6 kg), lower baseline SpO_2_ (94.6% vs. 96.6%), and more frequent baseline oxygen requirement (room air 41.9% in Q4 vs. 80.3% in Q1; face mask 22.2% vs. 2.1%). Radiographic severity and complications were more common in higher exposure groups, including multilobar involvement (49.2% in Q4 vs. 25.2% in Q1) and pleural effusion (16.9% vs. 7.1%). Median CRP was modestly higher in Q4 vs. Q1 [23.7 [14.2, 39.5] vs. 18.0 [10.8, 29.9] mg/L]. Hyponatremia prevalence increased from 26.5% (Q1) to 34.7% (Q4), and hypoalbuminemia increased from 16.4% to 24.5%. Bolus administration and vasoactive use were more frequent in Q4 (bolus 47.2% vs. 10.2%; vasoactive 12.5% vs. 0.8%). Isotonic maintenance fluids were used in 1,814/2,430 (74.7%) encounters and were similar across FMR quartiles (Table 1).

### Primary association between early IV fluids and respiratory deterioration

Respiratory deterioration rates increased across FMR quartiles from 3.1% (Q1) to 6.1% (Q2), 9.6% (Q3), and 21.2% (Q4) (Table 2). In unadjusted analysis, each 0.5-unit increase in FMR was associated with higher odds of deterioration (OR 1.88, 95% CI 1.69–2.08; *p* < 0.001); the association remained significant after multivariable adjustment (adjusted OR 1.45, 95% CI 1.28–1.65; *p* < 0.001). Compared with Q1, adjusted odds were higher in Q3 (adjusted OR 2.18, 95% CI 1.26–3.78) and Q4 (adjusted OR 3.56, 95% CI 2.08–6.10), with a significant ordinal trend. Overlap weighting comparing Q4 vs. Q1–Q3 also supported higher risk in the high-exposure group (OR 2.01, 95% CI 1.29–3.13; *P* = 0.002) (Table 2).

**Table 2:** Primary association between early IV fluids and respiratory deterioration (24–72 h).

Exposure	Events/*N* (%)	Unadjusted OR (95% CI)	*p* value	Adjusted OR (95% CI)	*p*_adj	FDR q (adj)
FMR (per +0.5 increase)	243/2430 (10.0%)	1.88 (1.69–2.08)	<0.001	1.45 (1.28–1.65)	<0.001	
FMR quartile: Q1 (lowest)	19/608 (3.1%)	1.00 (ref)		1.00 (ref)		
FMR quartile: Q2	37/607 (6.1%)	2.01 (1.14–3.54)	0.015	1.69 (0.95–3.00)	0.075	0.075
FMR quartile: Q3	58/607 (9.6%)	3.28 (1.93–5.57)	<0.001	2.18 (1.26–3.78)	0.005	0.008
FMR quartile: Q4 (highest)	129/608 (21.2%)	8.35 (5.08–13.72)	<0.001	3.56 (2.08–6.10)	<0.001	<0.001
*P* for trend (quartile as ordinal)				1.50 (1.28–1.75)	<0.001	
Overlap-weighted: Q4 vs. Q1–Q3				2.01 (1.29–3.13)	0.002	

### Dose–response analysis

**Figure 2:**
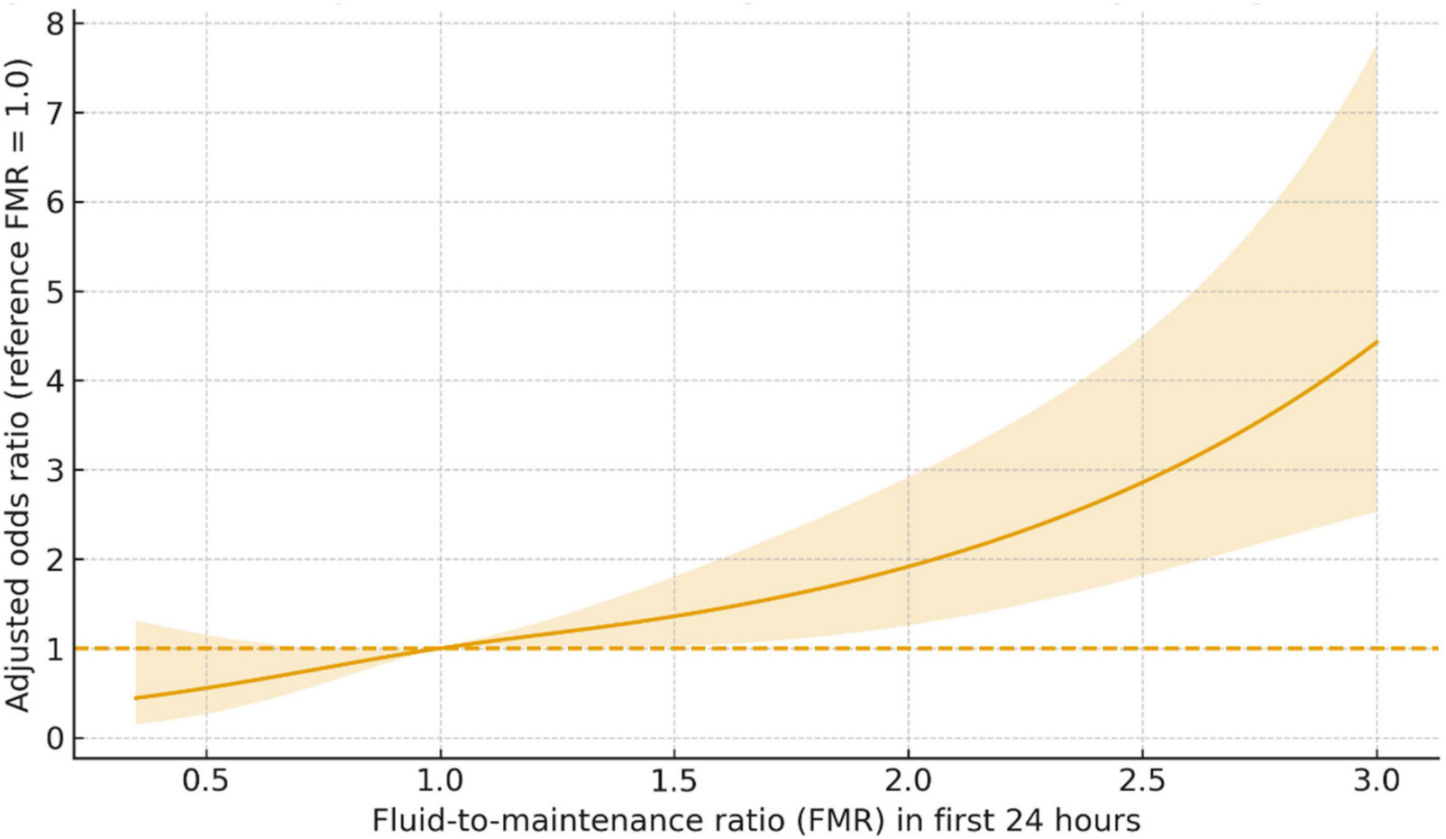
Adjusted dose–response between early IV fluids and respiratory deterioration. Adjusted odds ratio curve from a restricted cubic spline logistic model of FMR, referenced to FMR = 1.0. Shaded band indicates 95% CI.

In the adjusted restricted cubic spline model, odds of respiratory deterioration increased progressively with higher FMR relative to FMR = 1.0; uncertainty widened at exposure extremes where fewer observations contributed (Figure 2). The spline pattern was consistent with quartile-based results.

### Subgroup and sensitivity analyses

Across prespecified subgroups, associations were directionally consistent, and no interaction remained statistically significant after multiple-testing correction (Table 3). Findings were similar using 24–48 h and 24h–7d outcome windows and after excluding children receiving vasoactive agents (Table 4). Restricting to children without documented IV bolus attenuated the association but did not eliminate it (adjusted OR 1.29, 95% CI 1.05–1.58). Among children with analyzable 0–6 h infusion timing, median 0–6 h IV volume increased from 23.8 [18.5, 30.1] mL/kg in Q1 to 82.4 [67.0, 99.5] mL/kg in Q4, corresponding to 38.0% [31.0, 46.0] and 51.0% [43.0, 60.0] of the 24-hour total, respectively. In the anchored 0–6 h early-response subset with baseline and nearest-6-hour physiologic data (*n* = 220 events), median first-6-hour share of 24-hour IV volume was 44.0% [36.0, 54.0]. After additional adjustment for change in age-standardized heart rate, respiratory rate, systolic blood pressure, and diastolic blood pressure z scores, absolute change in SpO_2_, change in oxygen category, and first-6-hour fluid share, the association between FMR and later respiratory deterioration was attenuated but remained significant (adjusted OR per 0.5 FMR 1.27, 95% CI 1.10–1.47; *p* = 0.001), whereas first-6-hour fluid share itself was not independently associated after adjustment (OR per 10% increase 1.05, 95% CI 0.99–1.12; *p* = 0.108). In a complementary repeated-measures 0–6 h model using all available early vital-sign recordings and patient-specific time slopes (*n* = 231 events), results were similar (adjusted OR 1.29, 95% CI 1.12–1.49; *p* < 0.001) (Table 4). In the complete-case PCT subset (*n* = 1,326; 122 events), adding log(PCT) further attenuated the estimate and it was not statistically significant at *α* = 0.05 (adjusted OR 1.18, 95% CI 0.98–1.43; *p* = 0.079) (Table 4).

**Table 3:** Prespecified subgroup analyses (effect modification).

Subgroup	Level	Events/*N* (%)	Adjusted OR per +0.5 FMR (95% CI)	*p*_interaction	FDR q_interaction
Hyponatremia (Na <135)	No	146/1714 (8.5%)	1.45 (1.25–1.68)	0.923	0.923
Hyponatremia (Na <135)	Yes	97/716 (13.5%)	1.46 (1.20–1.79)		
Hypoalbuminemia (<35 g/L)	No	170/1969 (8.6%)	1.49 (1.29–1.73)	0.501	0.834
Hypoalbuminemia (<35 g/L)	Yes	73/461 (15.8%)	1.37 (1.12–1.69)		
Pleural effusion	No	183/2141 (8.5%)	1.49 (1.30–1.71)	0.359	0.834
Pleural effusion	Yes	60/289 (20.8%)	1.31 (1.01–1.69)		
Baseline oxygen (any vs. none)	No	77/1575 (4.9%)	1.37 (1.08–1.74)	0.417	0.834
Baseline oxygen (any vs. none)	Yes	166/855 (19.4%)	1.54 (1.33–1.78)		
Age <1 year	≥1y	148/1712 (8.6%)	1.47 (1.26–1.72)	0.757	0.923
Age <1 year	<1y	95/718 (13.2%)	1.42 (1.17–1.72)		

**Table 4:** Sensitivity analyses.

Sensitivity analysis	Events/*N* (%)	Adjusted OR per +0.5 FMR (95% CI)	*p* value
Primary (24–72 h; landmark cohort)	243/2430 (10.0%)	1.45 (1.28–1.65)	<0.001
Alternative window (24–48 h)	189/2430 (7.8%)	1.48 (1.29–1.70)	<0.001
Alternative window (24h–7d)	262/2430 (10.8%)	1.46 (1.29–1.65)	<0.001
Exclude vasoactive use	207/2310 (9.0%)	1.48 (1.30–1.70)	<0.001
Restrict to no bolus recorded	132/1855 (7.1%)	1.29 (1.05–1.58)	0.015
Complete-case PCT; adjust for log(PCT)	122/1326 (9.2%)	1.18 (0.98–1.43)	0.079
Anchored 0–6 h response model (age-standardized HR/RR/BP z scores)^a^	220/2214 (9.9%)	1.32 (1.15–1.51)	<0.001
Anchored 0–6 h response model + first-6 h fluid share^b^	220/2214 (9.9%)	1.27 (1.10–1.47)	0.001
Repeated-measures 0–6 h time-slope model^c^	231/2301 (10.0%)	1.29 (1.12–1.49)	<0.001

^a^ The anchored 0–6 h response model was limited to children with baseline and nearest-6-hour heart rate, respiratory rate, systolic blood pressure, diastolic blood pressure, SpO_2_, and oxygen-support data and additionally adjusted for change in age-standardized heart rate, respiratory rate, systolic blood pressure, and diastolic blood pressure z scores, absolute change in SpO_2_, and change in oxygen category.

^b^ This model further adjusted for the proportion of 24-hour IV volume delivered in the first 6 h.

^c^ The repeated-measures model was limited to children with ≥2 early vital-sign recordings within 6 h and incorporated patient-specific per-hour slopes for age-standardized heart rate, respiratory rate, systolic blood pressure, diastolic blood pressure, and SpO_2_ together with early oxygen-category trajectory.

## Discussion

In this study of children hospitalized with CAP, higher cumulative IV fluid exposure during the first 24 h was independently associated with a greater risk of subsequent respiratory deterioration between 24 and 72 h. Event rates increased from 3.1% in the lowest FMR quartile to 21.2% in the highest, and each 0.5-unit increase in FMR was associated with 45% higher adjusted odds of deterioration. These findings were supported by a graded exposure–response pattern, a significant trend across quartiles, and an overlap-weighted comparison of the highest-exposure group vs. the remainder.

The associations of higher cumulative IV fluid volume in the first 24 h with a graded increase in subsequent respiratory deterioration are mechanistically plausible because pneumonia-related inflammation can promote pulmonary capillary leak and edema formation, while the resolution of edema depends on intact alveolar epithelial transport. In acute lung injury (ALI) and acute respiratory distress syndrome (ARDS), alveolar fluid clearance is impaired in a large proportion of patients, predisposing to fluid accumulation when hydrostatic pressure rises or permeability increases (19). In systemic inflammatory states, endothelial glycocalyx injury further increases vascular permeability and can be worsened by hypervolemia, offering a biologic bridge between early fluid loading and worsening gas exchange (20). Clinically relevant correlates in our cohort also align with known CAP biology that hyponatremia in pediatric CAP frequently reflects inflammation-associated arginine vasopressin signaling and correlates with inflammatory biomarkers, while syndrome of inappropriate antidiuretic hormone secretion (SIADH) appears uncommon when strict criteria are applied (21). Similarly, hypoalbuminemia is common in pneumonia complicated by parapneumonic pleural effusion and tracks with effusion size, supporting a protein/fluid shift phenotype that may heighten susceptibility to respiratory worsening with additional IV fluids (22). Finally, the “early 24-hour” window is critical for stewardship because fluid decisions are front-loaded, and a 0–24 h exposure window temporally preceding outcome ascertainment strengthens causal interpretability while using maintenance-normalized metrics rooted in pediatric physiology (Holliday–Segar) improves age/weight comparability (23).

Compared with prior literature, our findings extend fluid-outcome concepts from adult critical illness into pediatric CAP. In adult ALI/ARDS, the ARDSNet FACTT trial demonstrated that a conservative fluid strategy improved lung physiology and increased ventilator-free days vs. a liberal strategy (without a significant mortality difference) (24). In adult septic shock and sepsis-induced hypotension, major contemporary trials (CLASSIC and CLOVERS) did not show mortality benefit for restrictive strategies but confirmed that “more fluid” is not universally superior once initial resuscitation is complete and that early (including 24-hour) fluid strategies are modifiable (25, 26). Pediatric infection data add an important cautionary perspective. The FEAST trial reported increased mortality with fluid boluses in African children with severe infection and impaired perfusion (27), and pediatric Surviving Sepsis Campaign guidance emphasizes smaller aliquots with frequent reassessment rather than fixed large-volume administration in pediatric septic shock (28). CAP-focused pediatric guidelines (e.g., PIDS/IDSA) prioritize diagnosis, severity assessment, and antimicrobials, offering relatively little granular evidence about optimal early IV fluid dosing in non-shock CAP phenotypes (29). Our study contributes by quantifying early IV fluid exposure relative to predicted maintenance and by separating exposure (0–24 h) from deterioration assessment (24–72 h).

The 2024 revision of the Chinese pediatric CAP guideline provides an up-to-date national framework for standardized CAP diagnosis and management in children's hospitals (30). Our data suggest a pragmatic complementary message that for hospitalized pediatric CAP patients without septic shock or clear hypoperfusion, clinicians should consider an early “maintenance-aware” IV fluid strategy, because early 24-hour exposure substantially above maintenance was associated with markedly higher risk of later respiratory deterioration. This is not an argument against resuscitation when shock is present, consistent with pediatric sepsis guidance (28), but it supports deliberate reassessment after initial stabilization, particularly in children with CAP phenotypes plausibly prone to third spacing or edema (21, 22). Because maintenance fluid choice can also worsen hyponatremia, adoption of isotonic maintenance IV fluids (with appropriate dextrose/potassium when indicated) is recommended for most children who require maintenance IVFs and may be especially relevant in CAP where hyponatremia is common (18). These results also motivate low-burden clinical decision support by automated calculation of 0–24 h IV mL/kg and FMR from weight and infusion totals, with alerts when exposure exceeds locally calibrated thresholds could operationalize early fluid stewardship in routine CAP care across Chinese pediatric wards and emergency settings.

Nevertheless, several limitations remain in our study. As in all observational fluid studies, residual confounding by indication is likely because clinicians administer more fluids to children who appear sicker, and unmeasured factors (e.g., perfusion assessments, clinician concern, subtle early respiratory trajectory) may influence both exposure and outcome. Our early-response analyses using age-standardized repeated vital-sign measures, blood pressure, and first 0–6 h fluid timing partially address the issue, but because time-updated physiological response can also mediate any harmful fluid effect, the attenuation seen in that model should not be interpreted as a definitive separation of confounding from causation. Total fluid exposure was incompletely characterized because oral intake, insensible losses, and net fluid balance were not fully captured, and the outcome definition may be affected by misclassification if escalation orders or ICU-transfer indications were inconsistently documented. Cardiac dysfunction may plausibly reduce fluid tolerance in CAP. Although we adjusted for documented congenital heart disease and early vasoactive use, standardized echocardiographic measures of ventricular function were not routinely available in the EHR and residual confounding by subclinical cardiac dysfunction may remain. In addition, IV fluid composition can influence serum sodium. While baseline sodium was defined using the earliest available measurement near presentation and isotonic maintenance fluid use was included in adjustment, we did not quantify total (enteral + parenteral) sodium delivery. Additionally, escalation thresholds can vary by clinician, staffing, and ward resources, introducing practice-pattern heterogeneity. The single-center design limits generalizability to other hospitals with different respiratory-support availability and admission practices. Future work should prioritize multi-center validation across diverse Chinese pediatric settings, prospective implementation of a fluid stewardship bundle, and integration of sodium-guided fluid strategies, including systematic monitoring and tailored tonicity/volume adjustments for children presenting with hyponatremia or high-risk phenotypes such as pleural effusion, to test whether modifying early IV fluid exposure can reduce preventable respiratory deterioration.

In conclusion, among children hospitalized with CAP, higher cumulative IV fluid exposure during the first 24 h was associated with a substantially increased risk of subsequent respiratory deterioration after the 24-hour landmark. These findings identify early IV volume as a clinically relevant, potentially modifiable exposure associated with worsening respiratory course, particularly when exposure exceeds maintenance by a large margin. Our results support a cautious, individualized “fluid stewardship” approach for most pediatric CAP patients and underscore the need for prospective, multicenter studies to confirm whether optimizing early IV fluids can reduce avoidable respiratory escalation.

## Data Availability

Data sets generated during the current study are available from the corresponding author on reasonable request.

## Supplementary material

Supplementary Figure S1 Study flow diagram.
