# Supplementary figures and images for: Cumulative intravenous fluid volume in the first 24 hours and risk of respiratory deterioration in children hospitalized with community acquired pneumonia
Source: Front Pediatr. 2026 Apr 10;14:1780766. doi: 10.3389/fped.2026.1780766 (PMC13106445; doi:10.3389/fped.2026.1780766)

**
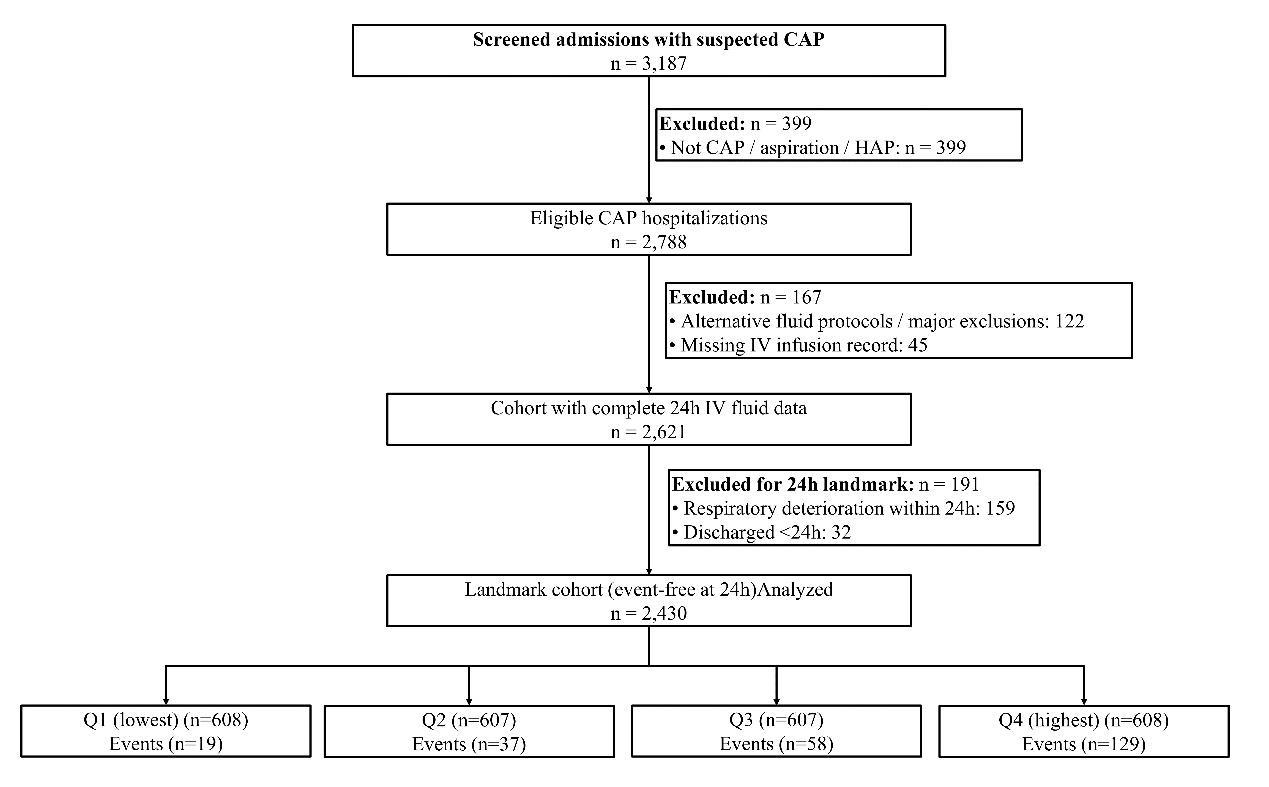
**

**Supplementary Figure S1. Study flow diagram.**

Supplement: Supplementary Figure S1 — Study flow diagram. [file Supplementaryfile1.docx]
